# The Early Signs of Measles

**Published:** 1908-07-04

**Authors:** 


### Diseases of Children.

### THE EARLY SIGNS OF MEASLES.

It is often a matter of the utmost importance to be able to forewarn parents or school teachers of the probable onset of measles. Unfortunately this disease appears especially infectious in its early stages, and the virus is communicated to other children before the individual is even suspected of being ill. Recently a case came under notice in which a child was in close association with other children on the day of onset without the infection being spread. In this instance the only sign of illness was a slight cough, which was put down to "getting a chill."

During the incubation period there is often a notable loss of weight, although the child appears in perfect health. This loss begins on the third to the fifth day. It is known as Meunier's sign, and may be of value in school outbreaks. Another sign, which is much more troublesome to elucidate than a mere loss in weight, is an increase in a polynuclear leucocytosis. According to Combe of Lausanne, it appears from one to two days before the symptoms. Brelet states that it reaches a maximum about six days before the rash appears. It then falls so much that during eruption, in uncomplicated cases, leucopenia is present.

A common mode of onset is one in which fever for twelve to twenty-four hours is associated with catarrhal signs, but the symptoms disappear and the child seems quite well. A diagnosis of acute coryza is made, the child is allowed to mix with others, and then after an interval of one to four days the ordinary signs of measles occur and a rash comes out. Be very suspicious of a febrile cold in a child coming apparently to a somewhat abrupt termination. Unusual drowsiness is quite a common initial symptom. In other cases there are fever and cough, due to faucial catarrh. Often some enlargement of the submaxillary lymph glands is also found. Here, too, the fever may subside, but the cough and faucial catarrh persist, and in a few days the temperature again rises, and the usual signs of measles develop.

Catarrh of the nose and eyes is a prominent symptom in most of the typical cases, but this is by no means a constant feature of the onset, and may not begin until the fourth day. Sometimes the stress of the catarrh falls on the larynx, and the child is thought to be suffering from acute laryngitis, of simple or diphtheric causation. Occasionally it is so severe as to necessitate tracheotomy. More commonly it is mild in severity and disappears as soon as the rash comes out.

One peculiarity about the catarrh of the eyes has not received sufficient attention. At the onset it is most marked over the bulbar conjunctiva, at the common site of phlyctenules. In the writer's experience the catarrh is in the horizontal plane, radiating outwards from the corneo-scleral junction. It may increase in severity or may subside before the appearance of the rash. This early limitation and distribution of the conjunctivitis often enables a differential diagnosis from simple conjunctivitis to be made with considerable confidence. More observations are required on this point.

Examination of the mouth and fauces may afford valuable information on the first day, and constantly does so before the rash comes out. The palate shows dark red patches of blotchy discolouration. This morbillary exanthem is said to appear the day before the rash, but it is often present earlier. The patches are more or less triangular or oval in shape, and best marked on the hard palate. At an earlier date there may be simply a little catarrh of the fauces, quite indistinguishable from a simple catarrh. Sometimes there is distinct redness and swelling of the mucous membrane of the cheeks and gums, a kind of erythemato-pultaceous stomatitis (Combe). The gums, especially those of the lower jaw anteriorly, are covered with an opalescent or whitish epithelial coating, thin and easily detached.

Personally, the writer attaches more importance to the bulbar conjunctivitis and the blotchy discolouration than to the so-called Koplik's spots. These buccal spots were first described by Flindt in 1880, and more fully by Filatow in 1895. Koplik drew special notice to them in 1896. They are almost always present from one to three days before the rash appears. Koplik described them as irregular, stellate or round, rose-coloured spots, with a bluish-white speck in the middle. It is better to regard them as quite minute white spots with a reddish areola, which may be absent at first, for it is this central speck which is the pathognomonic feature. They are best seen on that portion of the mucous membrane of the cheek which is opposite the lower molar teeth. This is the site of election, though they may be found on the buccal mucosa elsewhere, namely, lips, palate, uvula and fauces. In number they vary much, but are no indication of severity. They last for a few days and disappear at the height of the rash. In order to see them it is important to examine the mouth in good daylight, for they are liable to be overlooked in artificial light. A lens or short focus mirror is of assistance. Some writers have stated that the spots may be found in rötheln and even in normal mouths. This is probably due to a misconception of the characters of the spots, and to confusion with aphthae, the orifice of buccal glands, tiny particles of milk, mucus, etc.

Prodromal rashes are also described and are said to be quite common in children. According to J. D. Rolleston they appear on the first or second day, rarely after the onset of the catarrh, and precede buccal spots in half the cases. They may be as late as the third day, and they disappear before or merge occasionally into the true rash. The most common variety is a punctate erythema, general or patchy, and usually limited to the trunk and especially behind the ears. This type of rash simulates scarlatina. Other varieties are discrete papules, or isolated macules with blotches and irregular patches of confluent papules blotchy erythema, and urticaria. They generally disappear as the true rash comes out, and are of no prognostic value. They are both of value in diagnosis and a source of error.

Thus the early diagnosis of measles depends on the careful examination of the patient, with particular reference to the mouth, skin and conjunctiva, and a proper estimation of the importance of the coincidence of the special symptoms above mentioned. By careful attention to these above points it is frequently possible to prevent epidemic outbreaks of the disease in schools and hospital wards.

